# Evaluation of the diagnostic performance of the urine dipstick test for the detection of urinary tract infections in patients treated in Kenyan hospitals

**DOI:** 10.1099/acmi.0.000483.v3

**Published:** 2023-06-14

**Authors:** John Maina, John Mwaniki, Franklin Mwiti, Susan Kiiru, Japhet Katana, Fredrick Wanja, Joel Mukaya, Osborn Khasabuli, Benon Asiimwe, Stephen Gillespie, John Stelling, Stephen Mshana, Matthew Holden, Wilber Sabiiti, John Kiiru, HATUA consortium

**Affiliations:** ^1^​ Kenya Medical Research Institute, Centre for Microbiology Research, Nairobi, Kenya; ^2^​ Jomo Kenyatta University of Agriculture and Technology, Juja, Kenya; ^3^​ Kentyatta National Hospital, Nairobi, Kenya; ^4^​ Makerere University, Kampala, Uganda; ^5^​ University of St Andrews, School of Medicine, St Andrews, UK; ^6^​ Department of Medicine, Brigham and Women’s Hospital and Harvard Medical School, Boston, MA, USA; ^7^​ Catholic University of Health and Allied Sciences, Mwanza, Tanzania; ^8^​ Ministry of Health, Nairobi, Kenya

**Keywords:** dipstick test, urine culture, urinary tract infections, nitrites, leucocytes esterase

## Abstract

**Introduction.:**

Culture is the gold-standard diagnosis for urinary tract infections (UTIs). However, most hospitals in low-resource countries lack adequately equipped laboratories and relevant expertise to perform culture and, therefore, rely heavily on dipstick tests for UTI diagnosis.

**Research gap.:**

In many Kenyan hospitals, routine evaluations are rarely done to assess the accuracy of popular screening tests such as the dipstick test. As such, there is a substantial risk of misdiagnosis emanating from inaccuracy in proxy screening tests. This may result in misuse, under-use or over-use of antimicrobials.

**Aim.:**

The present study aimed to assess the accuracy of the urine dipstick test as a proxy for the diagnosis of UTIs in selected Kenyan hospitals.

**Methods.:**

A hospital-based cross-sectional method was used. The utility of dipstick in the diagnosis of UTIs was assessed using midstream urine against culture as the gold standard.

**Results.:**

The dipstick test predicted 1416 positive UTIs, but only 1027 were confirmed positive by culture, translating to a prevalence of 54.1 %. The sensitivity of the dipstick test was better when leucocytes and nitrite tests were combined (63.1 %) than when the two tests were separate (62.6 and 50.7 %, respectively). Similarly, the two tests combined had a better positive predictive value (87.0 %) than either test alone. The nitrite test had the best specificity (89.8 %) and negative predictive value (97.4 %) than leucocytes esterase (L.E) or both tests combined. In addition, sensitivity in samples from inpatients (69.2 %) was higher than from outpatients (62.7 %). Furthermore, the dipstick test had a better sensitivity and positive predictive value among female (66.0 and 88.6 %) than male patients (44.3 and 73.9 %). Among the various patient age groups, the dipstick test’s sensitivity and positive predictive value were exceptionally high in patients ≥75 years old (87.5 and 93.3 %).

**Conclusion.:**

Discrepancies in prevalence from the urine dipstick test and culture, the gold standard, indicate dipstick test inadequacy for accurate UTI diagnosis. The finding also demonstrates the need for urine culture for accurate UTI diagnosis. However, considering it is not always possible to perform a culture, especially in low-resource settings, future studies are needed to combine specific UTI symptoms and dipstick results to assess possible increases in the test’s sensitivity. There is also a need to develop readily available and affordable algorithms that can detect UTIs where culture is not available.

## Data Summary

Data regarding the nitrite test, leucocyte esterase, blood and white cell count were entered into the Epicollect data management tool. An Excel sheet was generated from Epicollect (uploaded as supplementary materials, available in the online version of this article) and exported to SPSS to calculate prevalence, sensitivity, specificity, and positive and negative values using urine culture results as the gold standards. Graphs were generated with Microsoft Excel.

## Introduction

Urinary tract infections (UTIs) are among the most common community-acquired bacterial infections globally [1]. While most UTIs are associated with mild and brief morbidity characterized by dysuria, lower abdominal pain and frequent urination, these symptoms are a nuisance to many. Furthermore, if not treated promptly, the disease can progress to cause a more severe infection in the kidney. Despite a timely and accurate diagnosis of UTI being the baseline for disease management, most healthcare facilities in low- and middle-income countries (LMICs) lack the capacity necessary to conduct culture, the gold standard test [2]. Therefore, treatment of UTIs in these countries is often based on symptoms and results of urine dipstick tests [3]. The urine dipstick method is easy and inexpensive to perform. It produces results quickly (approximately 5 min), making it popular in many hospitals in Kenya, as in many LMICs. Unfortunately, most healthcare facilities in Kenya do not have a microbiology laboratory, nor do they have the capacity to perform urine cultures. The logistics of setting up such facilities, especially in grass root healthcare facilities that are often the first contact point for many patients, are problematic due to a lack of infrastructure capacity. The technical aspect of test performance, the complexity of analysis and the long turnaround time mean that results are rarely available in a timeframe to affect patient management decisions. Despite these considerable technicalities and challenges in culture performance for accurate diagnosis, the continued use and overreliance on dipstick screening remains a significant concern due to its inability to accurately detect and discriminate active UTI cases.

In dipstick diagnostic tests for UTIs, leucocyte esterase (L.E) and nitrites (N) are the essential test parameters [4]. Leucocyte esterase enzymes are released by white blood cells (WBCs) and are an excellent quantitative measure of WBCs in the urinary tract [5]. On the other hand, nitrites (N) detect the presence of bacteria that can convert nitrates to nitrites, especially those belonging to the family *Enterobacteriaceae* [6]. In addition to leucocyte esterase and nitrites, other parameters such as red blood cells (RBCs), proteins and pH can reinforce the UTI dipstick test [5] For instance, a pH of ≥7.2, though not conclusive, could indicate the presence of urease-producing organisms such as *Klebsiella* and *Proteus* spp. [7]. The presence of urease enzymes in these bacteria leads to ammonia production, which leads to pH elevation [8]. Urine pH depends on diet and, therefore, is not as helpful in the absence of other tests such as arterial blood gas (ABG) and metabolic panels. The presence of blood in the urine may also suggest a bleeding urinary tract due to an active UTI. Additionally, several other factors, including genital and urinary malignancy, kidney stones, nephritic syndrome, huge muscle breakdown (rhabdomyolysis) [5], and contamination with semen and menses in women, may lead to a positive RBC test. Positive protein urine may also suggest post-renal proteinuria, with more than 60 % of such cases being positive for UTIs. The present study therefore sought to evaluate the utility of urine dipstick tests in UTI diagnosis compared to the standard culture method.

## Methodology

### Study overview

The present study was part of the large ‘HATUA Consortium’. HATUA is an East African consortium that used a mixed methods research design to holistically determine drivers of antimicrobial resistance using UTI as the flag disease. This 5 year study was conducted between 2018 and 2022 in Kenya, Uganda and Tanzania (https://gtr.ukri.org/projects?ref=MR%2FS004785%2F1). A subset of the study’s objectives was to evaluate the diagnostic performance of the urine dipstick test as a proxy for UTI diagnosis using culture as the gold standard.

### Study design

To evaluate the accuracy of the urine dipstick test for UTI diagnosis, a cross-sectional hospital-based study design was used to recruit 1898 patients using a purposive sampling approach.

### Participant recruitment

Participants presenting with abdominal pain, burning sensation, discharge, frequent urination and/or fever symptoms were recruited for the study by doctors and clinical officers at various healthcare facilities in Kenya. Potential participants who failed to meet the empiric UTI diagnosis criteria determined by the study doctor and who did not consent were excluded.

### Study site

**Fig. 1.**
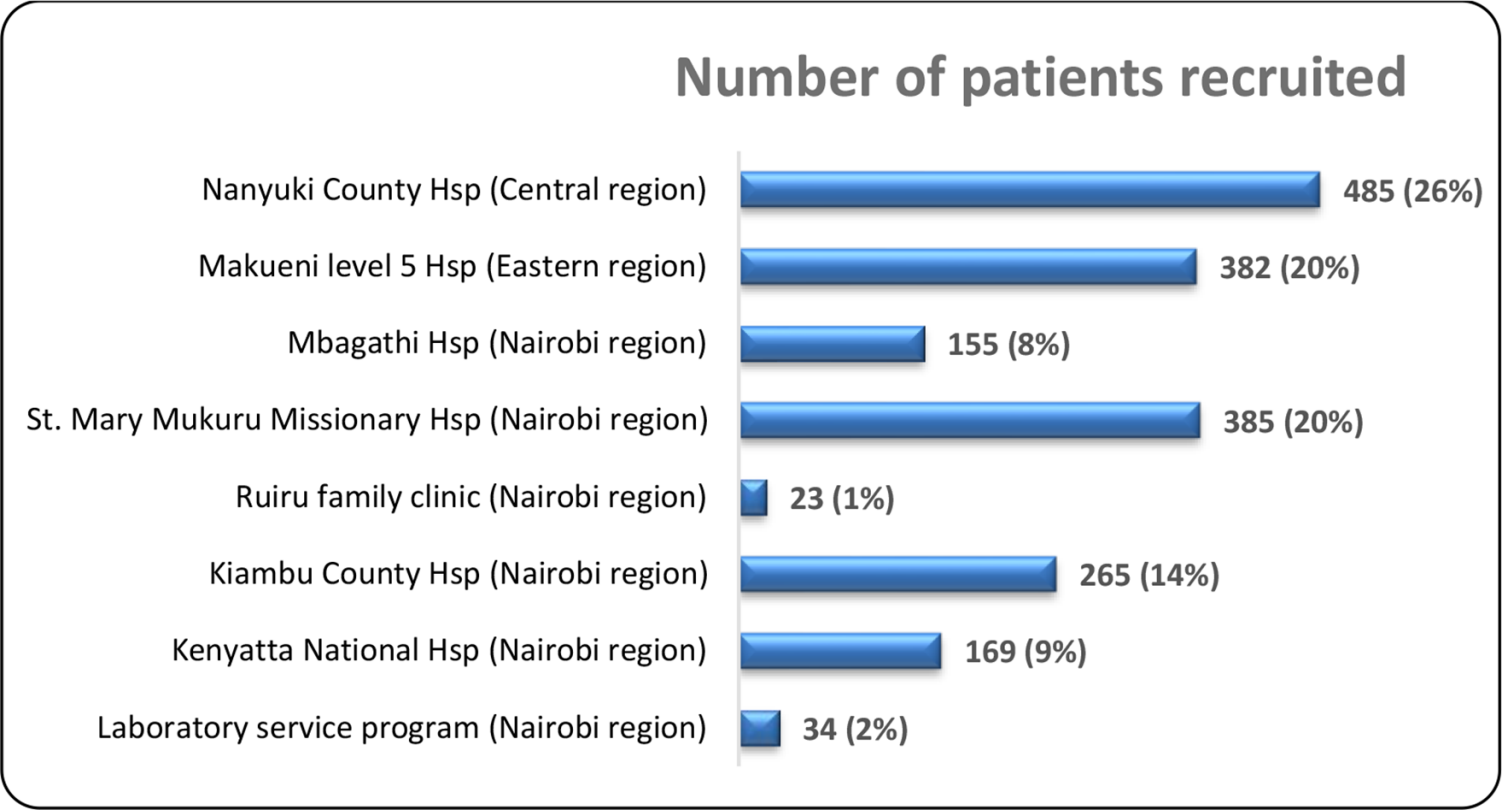
Details of patients recruited for the present study. The HATUA study Kenya chapter had three sampling regions: former Nairobi, Central region, and Eastern province. The study recruited 1,898 patients in eight healthcare facilities within the three regions. The Nairobi region had the highest number of patients recruited (54%), followed by Central (26%) and Eastern (20%).

The study was implemented in eight healthcare facilities in the Central, Eastern and Nairobi regions of Kenya (Fig. 1).

### Sample processing

Approximately 10 ml of a midstream urine sample was collected in a sterile container and processed within 10 min at the study sites. A urine culture was done before dipping the urine stick to prevent contamination. The strip (ACON Laboratories) was dipped into urine and immediately removed, followed by a wait period of 1–2 min (according to the manufacturer’s instructions). Although conventionally RBCs are not considered in the UTI dipstick test, we screened for them to assess their usability. L.E and RBCs were recorded as negative, trace, 1+ (small), 2+ (moderate) or 3+ (large), while nitrites (N) were recorded as negative or positive. A test was considered positive when the specimen presented N or L.E activity greater than or equal to a trace amount of leucocytes.

All urine samples, including those with negative dipstick test screening, were cultured on cysteine lactose electrolyte-deficient (CLED), blood agar (BA) and mannitol salt ager (MSA) (Oxoid) using the quadrant culture method and incubated at 37 °C for 18–24 h. Testing all samples collected was crucial in detecting UTI cases that may have been missed by dipstick test screening. A monoculture growth with a colony count of ≥10^4^ c.f.u. ml^−1^ was treated as a confirmed positive UTI case, while a culture with more than one colony type and growth of <10^4^ c.f.u. ml^−1^ was considered a contaminant [9]. Contaminated cultures or those without microbial growth were classified as negative for UTI.

### Ethics statement

Prior to the study’s commencement, ethical approval was sought from the Kenya Medical Research Institute Scientific Ethical Review Committee (No. KEMRI/SERU/CMR/P00112/3865), National Commission for Science, Technology and Innovation (NACOSTI) and Health facilities, included in the study. The study and its intended objectives were introduced to the participants at the study site, and written consent was obtained voluntarily. Assent and consent were obtained for participants aged 13–17 years, while only consent was obtained for the guardians of participants younger than 13 years. Married participants under the age of 18 years, were deemed to be minor adults, capable of consenting.

## Results

### Dipstick and culture results

This study recruited 1707 (89.9 %) adult outpatients, 123 (6.5 %) adult inpatients, 47 (2.5 %) child outpatients and 21 (1.1 %) child inpatients. The minimum age of the patients was 1 year, the maximum was 102 years, and the mean was 30.7 years with a median of 28 years. The study analysed 1898 urine samples from in- and outpatients of all ages. Nitrite and leucocyte tests together predicted UTI positivity in 1416 of 1 898 samples. However, culture confirmed 1027 of 1898 suspected UTI cases, translating to a test population prevalence of 54.1 % (Table 1). A total of 133 (27.6 %) predicted to be UTI-negative by dipstick test were positive by culture, while 36.9 % predicted to be positive were either contaminants or showed no growth on the medium.

**Table 1.** Comparative analysis of the dipstick test and culture with regard to accuracy of UTI diagnosis

		UTI status by culture			Total
		Contaminants	No growth	UTI	
UTI status by dipstick	Negative	105	244	133	482
		21.8 %	50.6 %	27.6 %	
	Positive	182	340	894	1416
		12.9 %	24.0 %	63.1 %	
Total		287	584	1027	1898
		15.1 %	30.8 %	54.1 %	

Table 1 assesses the accuracy of the dipstick screening test in detecting UTIs against theculture-based method.

### Urine dipstick test sensitivity and specificity

**Fig. 2.**
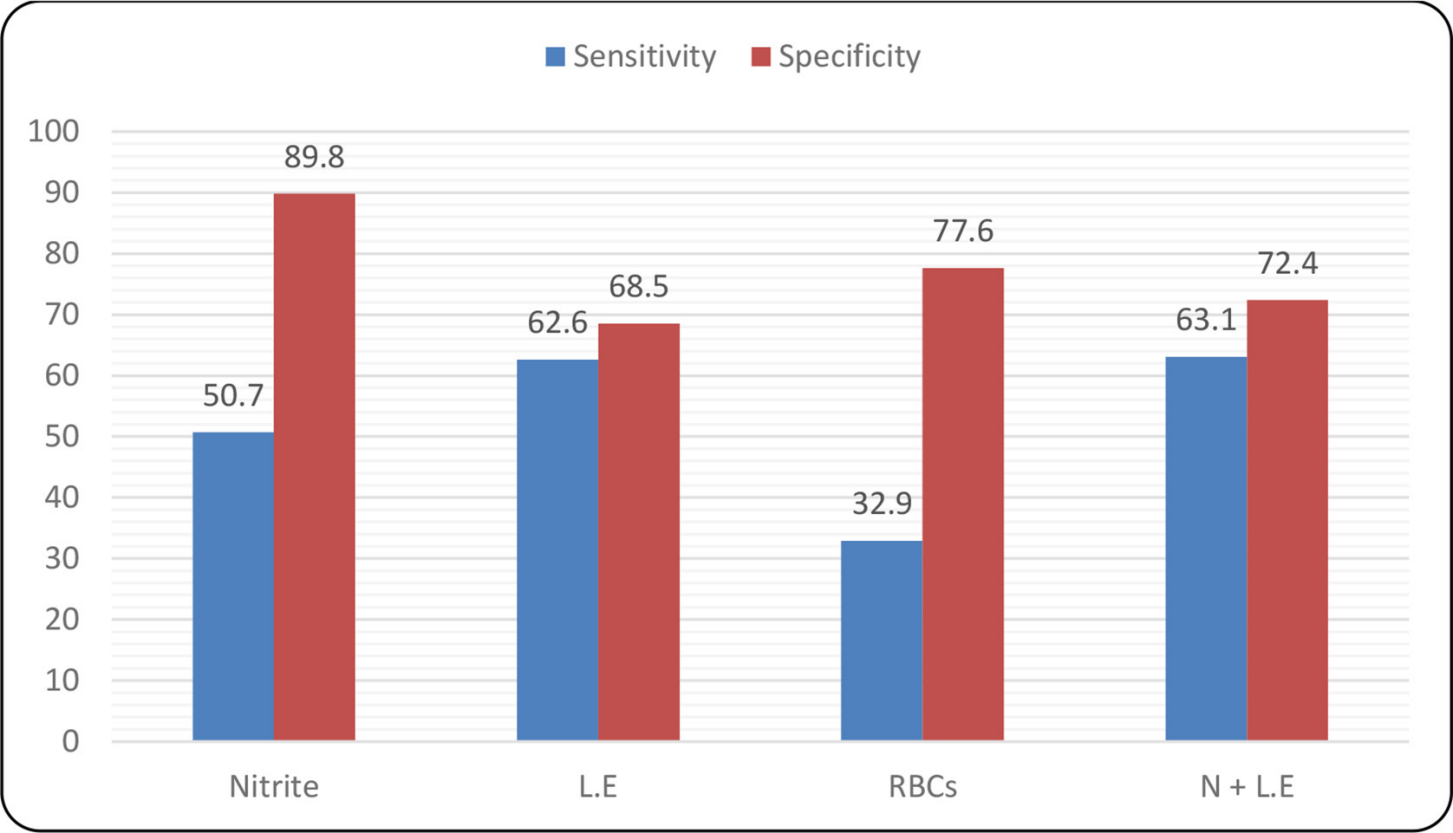
Represent sensitivity and specificity scores for various dipstick test parameters vital inUTI screening. A high sensitivity score indicates that a test can accurately identify a positive case,while a high specificity score indicates that it can accurately identify a negative case.

The nitrite test had the lowest ability to accurately detect a positive UTI case (sensitivity, 50.7 %). Simultaneously, the nitrite test’s specificity (89.8 %) was higher than that of the leucocyte esterase test (68.5 %) and the combined results of the two tests (72.4 %). The specificity of the dipstick test was better when leucocytes and nitrite tests were combined (63.1 %) than when the two tests were separate (Fig. 2). The RBC test for UTI screening had the lowest sensitivity at 32.9 %.

### Positive and negative predictive value of UTI screening with the dipstick test

**Fig. 3.**
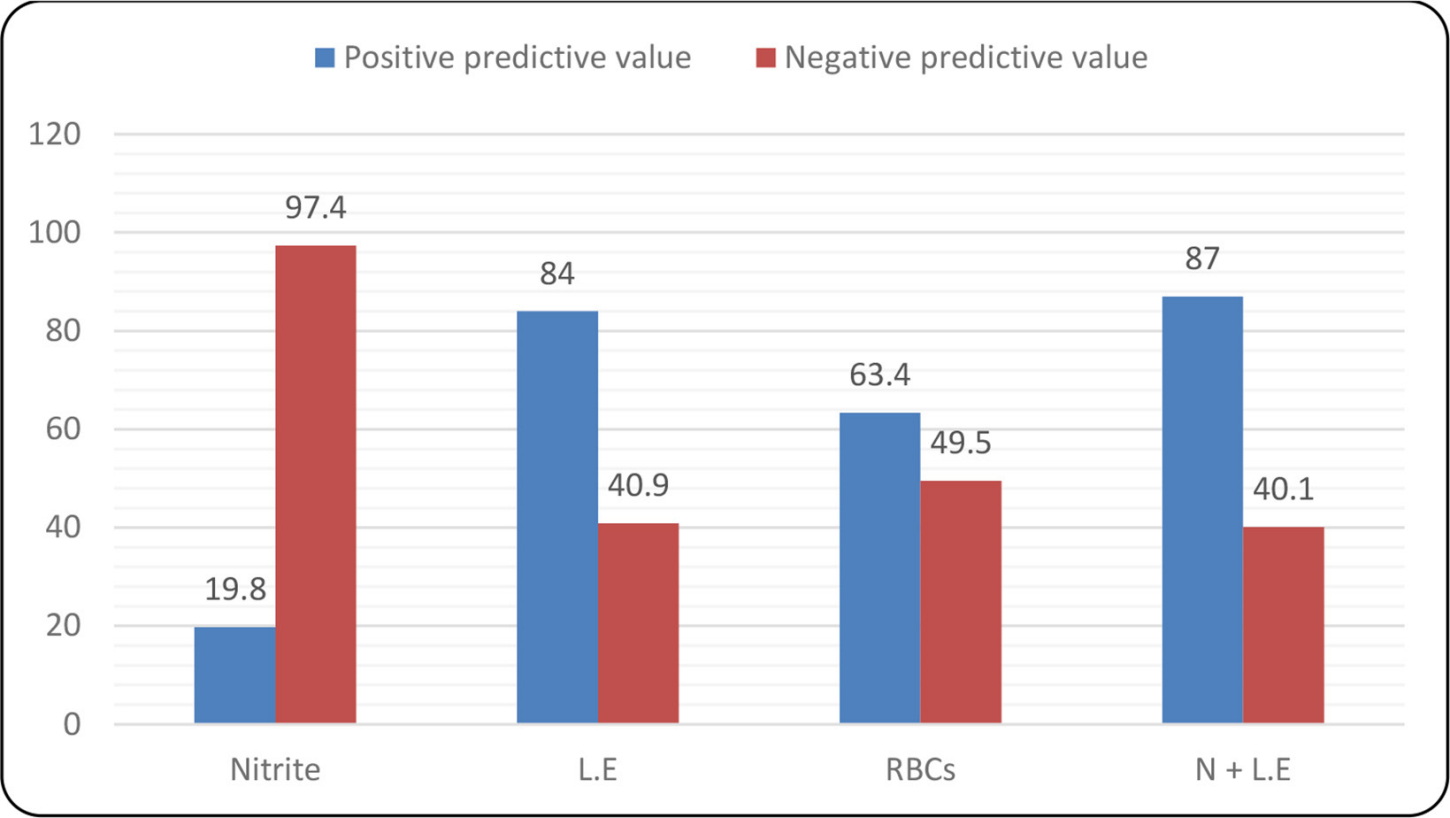
Shows positive and negative predictive values for various dipstick test parametersvital in UTI screening using the dipstick test. The predictive values indicate the tests’ ability todetermine the true result predicted as positive or negative. A high value indicates a better ability, whilea low indicates poor ability.

The nitrite test had the lowest analytical ability to detect a positive UTIs accurately, with a positive predictive value (PPV) of 19.8 % (Fig. 3). However, the nitrite test had a better ability to accurately detect a true negative UTIs (97.4 %) than either the leucocyte test alone (40.9 %) or both tests combined (40.1 %). On the other hand, the leucocyte and nitrite tests combined had an enhanced ability to accurately detect a positive UTI (87 %) than either test alone. The leucocyte test had a higher PPV (84 %) than the nitrite test (19.8 %). The RBC test had a better PPV than the nitrite test (40.9 % vs. 19.8 %) and better negative predictive value (NPV) than the L.E test (49.5 % vs. 40.9 %).

### UTI diagnosis analysis across study variables

The dipstick test based on either the nitrite or L.E test had a better sensitivity in samples from inpatients (69.2 %) than from outpatients (62.7 %; Table 2). However, the specificity and ability of the dipstick test to accurately predict a positive test were better in samples from outpatients (73.9 and 88.1 %, respectively) compared with inpatients (60.4 and 75.0 %). The dipstick test was better able to detect UTIs among child patients (inpatients at 90.1 % and outpatients at 70.7 %) than among adult patients (inpatients at 66.3 % and outpatients at 62.5 %). On the other hand, the test had a better sensitivity and PPV among female (66.0 and 88.6 %) patients compared to their male counterparts (44.3 and 73.9 %). However, the test specificity and NPV were better among the male (82.2 and 56.5 %) than the female patients (67.4 and 33.9 %). Among the various patient age groups, sensitivity and PPV of the dipstick test were exceptionally high in patients ≥75 years old (87.5 and 93.3 %) and those aged 65–74 years (74.2 and 100 %). However, despite a specificity of 100 % among those aged 65–74 years, the dipstick test had a low ability to accurately detect a true negative (33.3 %) in this age group. Table 2 details the dipstick test’s sensitivity, specificity, and PPV and NPV across the various study variables.

**Table 2.** Sensitivity, specificity, and positive and negative predictive values for the dipstick test across the various study variables

Variable		*N*	UTI status		Sensitivity	Specificity	Positive predictive value	Negative predictive value
			Negative	Positive				
Patient category	Inpatient	144	60	84	69.2	60.4	75	53.3
	Outpatient	1754	811	943	62.7	73.9	88.1	39.1
Patient type	Adult inpatient	123	54	69	66.3	62.8	76.8	50
	Adult outpatient	1707	796	911	62.5	74.2	88	39.4
	Child inpatient	21	6	15	90.1	50	66.7	83.3
	Child outpatient	47	15	32	70.7	50	90.6	20
Gender	Female	1550	634	916	66	67.4	88.6	33.9
	Male	348	237	111	44.3	82.2	73.9	56.5
Age group (years)	<18	79	28	51	71.9	54.5	80.4	42.9
	18–24	493	207	286	64.9	71.3	90.6	32.4
	25–34	837	401	436	60.7	72.9	86.7	38.9
	35–44	294	146	148	63.1	74.7	83.1	50.7
	45–54	96	48	48	58.6	73.1	85.4	39.6
	55–64	44	24	20	60	73.4	75	58.3
	65–74	35	12	23	74.2	100	100	33.3
	≥75	20	5	15	87.5	75	93.3	60

Table 2 shows assesses the dipstick test‘s accuracy in urinary tract infections (UTI) diagnosis across thevarious patients’ variables against the gold standard, culture. Both nitrite or positive tests were used to calculate the tests’ sensitivity, specificity, PPV, and NPV. Values for Sensitivity, Specificity,Positive predictive value, and Negative predictive value are expressed as percentages.

### UTI prevalence estimates based on dipstick test parameters

**Fig. 4.**
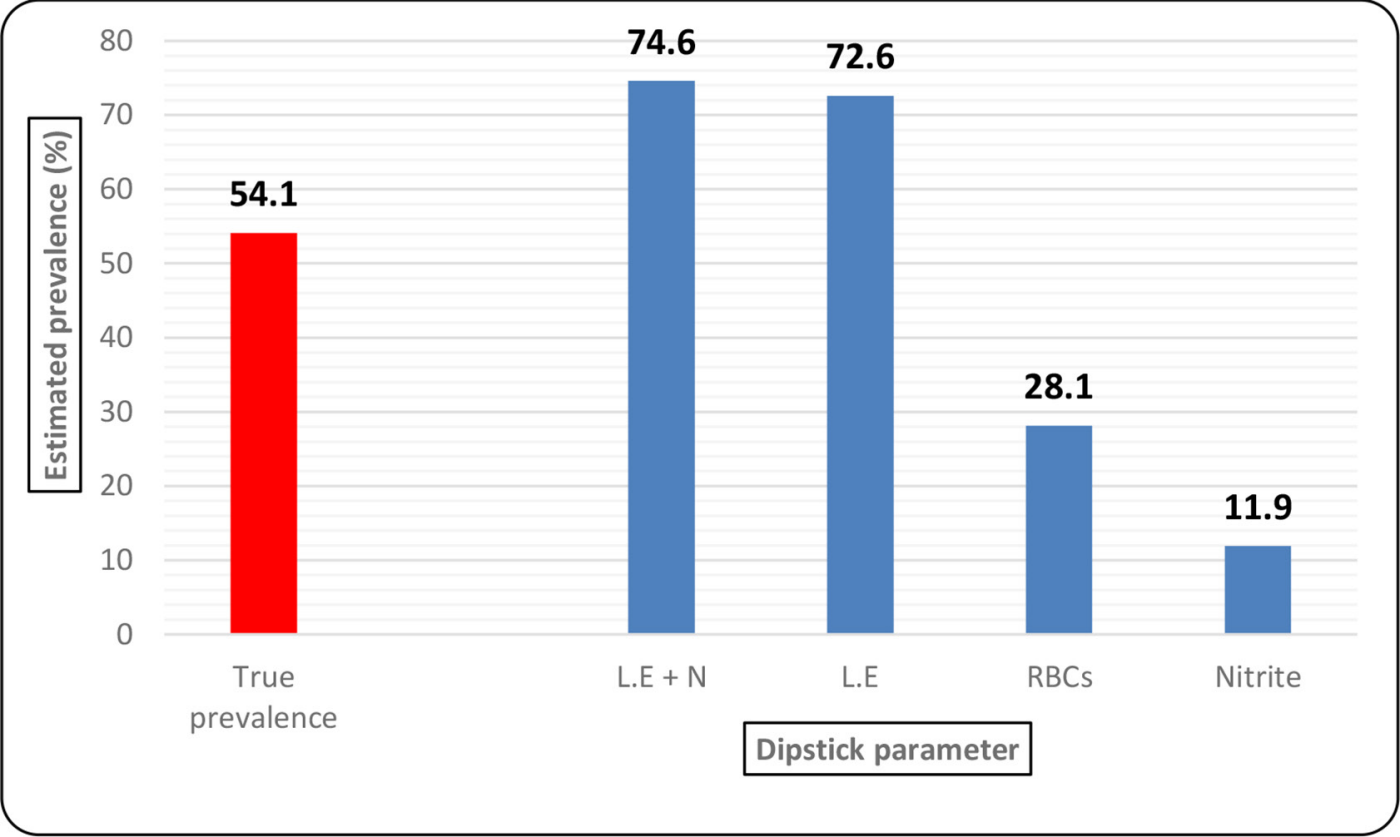
Estimated prevalence with the nitrate, L.E, RBC and N+L.E tests

When either L.E or nitrite positivity was used to screen for UTIs, a total of 1416 tests were deemed positive, translating to an estimated prevalence of 74.6 %, which is higher than the 54.1 % true prevalence. The L.E test (72.6 %) also overestimated the prevalence. Nitrite (11.9 %) and RBC test (28.1 %) prevalence were less than the actual prevalence (Fig. 4).

## Discussion

Whereas the dipstick test is a readily available and cheap proxy test for UTI diagnosis, variability raises concerns about its accuracy in UTI diagnosis. For instance, the present study’s dipstick sensitivity and specificity of 63.1 and 72.4% differ from the 51 and 83% reported by Bhansali [10] in a similar study in India [10]. Similarly, positive (65 %) and negative (73 %) predictive values in Bhansali’s (2020) study also differ from the 87.0 and 40.1 % recorded in our study. The variability in the dipstick UTI screening test can be caused by the patient selection criteria, urine type and time of sample colllection, the experience of the lab staff, and the time taken to read the results [11].

The nitrite test has also shown variability across studies. According to Ballazreg *et al*., the nitrite test had a sensitivity of 48 % and specificity of 95 %, which varies from 50.9 and 89.8 % in the present study [12]. In the present study, the nitrite test had the lowest sensitivity compared to the leucocyte esterase (L.E) (50.7 % vs. 62.6 %) test and both tests combined (50.7 % vs. 63.1 %). In addition, the nitrite test also had the lowest PPV (19.8 %), which mirrored the findings of Ballazreg’s study. Gram-positive bacteria do not produce nitrite reductase enzyme, making the nitrite test unreliable in detecting UTIs caused by these bacteria. Furthermore, the decreased excretion of nitrites in urine lowers pH, which can also cause a low sensitivity for the nitrite test, which explains the present study’s findings [13]. The sensitivity of the nitrite test may also be impacted by the time of sample collection. The conversion of nitrate to nitrite (nitrification) requires time (at least 4 h) and therefore a midsream sample collected during the first void in the morning is likely to yield a better sensitivity [14]. Therefore, this could also explain the recorded low sensitivity of the nitrite test, considering the present study did not have a standard sample collection time. The nitrite test’s low sensitivity shows that this test alone is insufficient in screening for possible UTIs. By contrast, the nitrite test has been shown to have better specificity and NPVs, a finding reflected in the current study (89.8 and 97.4 %) [11, 12]. These results indicate that the nitrite test is a better parameter detecting true negative UTIs caused by Gram-negative bacteria in the absence of a culture test.

Often, the L.E test has a better sensitivity than the nitrite test. The L.E sensitivity of 57.6 % reported by Prah *et al*. [15], 92 % by Suresh *et al*. [16] and 62.7 % by Mohanna *et al*. [17] were all higher than the nitrite test, which is similar to the present study [15–17]. Most infections elicit an immune response and production of WBCs, which could explain why leucocyte esterase was a better UTI predictor. Nonetheless, nitrite and L.E tests combined were a better UTI predictor, with the highest sensitivity and PPV than either of the separate tests. The relatively low sensitivity of the L.E test may be attributed to factors such as low bacterial counts in urine, elevated specific gravity and glycosuria [18].

Dipstick test results have also been shown to vary in the different study populations. For instance, in the elderly population, the dipstick test is deemed unreliable and often leads to elevated false positive UTI diagnoses [19]. This could explain why the dipstick test had an over 90 % PPV in patients older than 65 years. Therefore, the dipstick test can be misleading in empirical treatment of the elderly, as asymptomatic bacteria are common in these patients and may not require antibiotic treatment [20]. On the other hand, the dipstick test has been found to be comparatively more reliable in the middle-aged population [21]. This is contrary to the present study’s findings, which indicates dipstick test performance was better in patients >75 years old with sensitivity and PPV of 87.5 and 93.3% compared to 63.1 and 83.1 % in patients between 35 and 44 years. The dipstick test is also considered to be unreliable in UTI diagnosis in children due to sample contamination and the inability to allow adequate incubation time for nitrate-to-nitrite conversion [22]. This could explain the comparatively low specificity and NPV of 54.5 and 42.9 % in participants below 18 years. Dipstick screening was more sensitive in female patients (66.0%) but more specific in males (82.2%), and similar results have been noted in previous studies [12, 23].

The blood cells test as a predictor of UTI had a sensitivity of 32.9 %, lower than the 63.9 % reported by Mambatta *et al*. [24] [24]. In addition, the RBC test had the lowest sensitivity at 32.9 %, which indicates that RBCs represent a poor indicator of active UTIs. This confirms that the array of confounding factors of haematuria makes RBC screening in urine unreliable for UTI diagnosis.

The prevalence of UTI based on dipstick parameters (74.6 %) was higher than the 54.1 % observed by the gold standard. The discrepancy in prevalence was also observed when the nitrite test (11.9 %) and leucocyte esterase (72.6 %) were used separately. The above findings align with those of Deepthi *et al*., who reported UTI prevalence of 21.15%, 30.76%, 36.53% and 38.46 % by the nitrite, L.E, culture and dipstick tests, respectively, in a study in India [25]. Although not within the scope of the current study, over-estimation of UTI prevalence means incorrect diagnoses and unnecessary prescriptions to treat UTI-like symptoms. The dipstick test UTI misdiagnosis could be attributed to factors such as patients’ antimicrobial medication at the time of sampling, urinary tract inflammation or sexually transmitted infection [26].

## Conclusion

A combination of relevant dipstick parameters overestimate the prevalence of UTIs. This could result in unnecessary prescription and over-prescription of antimicrobials. On the other hand, using crucial urine dipstick parameters for UTI diagnoses, such as nitrite, as demonstrated in the present study, could lead to UTI underestimation and under-prescription. The findings therefore strongly imply the need to perform urine culture for accurate UTI diagnosis. Thus, further research using emerging rapid UTI culture methods is required. However, the practicality of performing culture in settings that lack the infrastructure capacity makes accurate UTI diagnosis a challenge. Similar future studies can combine specific UTI symptoms and dipstick results to assess possible increases in the test’s sensitivity. There is also a need to develop readily available and affordable algorithms that can detect UTIs where culture is not available.

## Strength of the study

The large sample size used in the study allows for a more precise prevalence estimate. In addition, the culture of all urine samples allows for more precise estimation and detection of possible UTIs that the dipstick test screening would otherwise miss.

## Study limitations

Distinguishing between a true UTI pathogen and a contaminant in a polymicrobial culture presents a major challenge and may lead to biased interpretation of the results. Despite a patient having a positive urine dipstick test, antibiotic use may suppress bacterial growth, resulting in a negative result or growth below the UTI threshold. In the present study, antimicrobial intake at the time of recruitment was not an exclusion factor, which may have led to disparities in UTI prevalence by dipstick and culture.

## Supplementary Data

Supplementary material 1
